# Ascending midbrain dopaminergic axons require descending GAD65 axon fascicles for normal pathfinding

**DOI:** 10.3389/fnana.2014.00043

**Published:** 2014-06-05

**Authors:** Claudia M. García-Peña, Minkyung Kim, Daniela Frade-Pérez, Daniela Ávila-González, Elisa Téllez, Grant S. Mastick, Elisa Tamariz, Alfredo Varela-Echavarría

**Affiliations:** ^1^Departamento de Neurobiología del Desarrollo y Neurofisiología, Instituto de Neurobiología, Universidad Nacional Autónoma de MéxicoQuerétaro, México; ^2^Department of Biology, University of NevadaReno, NV, USA

**Keywords:** axon interaction, fasciculation, dopaminergic, nigrostriatal pathway, NCAM, Robo

## Abstract

The Nigrostriatal pathway (NSP) is formed by dopaminergic axons that project from the ventral midbrain to the dorsolateral striatum as part of the medial forebrain bundle. Previous studies have implicated chemotropic proteins in the formation of the NSP during development but little is known of the role of substrate-anchored signals in this process. We observed in mouse and rat embryos that midbrain dopaminergic axons ascend in close apposition to descending GAD65-positive axon bundles throughout their trajectory to the striatum. To test whether such interaction is important for dopaminergic axon pathfinding, we analyzed transgenic mouse embryos in which the GAD65 axon bundle was reduced by the conditional expression of the diphtheria toxin. In these embryos we observed dopaminergic misprojection into the hypothalamic region and abnormal projection in the striatum. In addition, analysis of Robo1/2 and Slit1/2 knockout embryos revealed that the previously described dopaminergic misprojection in these embryos is accompanied by severe alterations in the GAD65 axon scaffold. Additional studies with cultured dopaminergic neurons and whole embryos suggest that NCAM and Robo proteins are involved in the interaction of GAD65 and dopaminergic axons. These results indicate that the fasciculation between descending GAD65 axon bundles and ascending dopaminergic axons is required for the stereotypical NSP formation during brain development and that known guidance cues may determine this projection indirectly by instructing the pathfinding of the axons that are part of the GAD65 axon scaffold.

## Introduction

The Nigrostriatal pathway is formed by axons of A9 dopaminergic (DA) (Dahlstroem and Fuxe, 1964) from the Substantia Nigra pars compacta (SNpc) located in the ventral midbrain. DA axons navigate rostrally from the midbrain through the ventral aspect of the diencephalon as part of the medial forebrain bundle (MFB) along with other midbrain dopaminergic axons from the ventral tegmental area (A10) and the retrorubral nucleus (A8) (Albanese et al., 1986). In the rostral diencephalon, A9 axons veer into the striatal anlage forming the mesostriatal pathway while A10 axons continue to the cortex forming the mesocortical pathway.

Several chemotropic axon guidance systems have been implicated in the pathfinding of midbrain dopaminergic axons. These include Ephrins, Slits, Netrin1, Sonic hedgehog (Shh), Class 3 Semaphorins (Sema), and Wnt5a (reviewed in Blakely et al., 2013). The expression of Robo proteins, Neuropilins (Npns), and DCC, which are the receptors for Slits, class III Semaphorins, and Netrin1, respectively, has been detected in embryonic DA neurons (Lin et al., 2005; Lin and Isacson, 2006; Hernandez-Montiel et al., 2008; Tamariz et al., 2010; Xu et al., 2010; Dugan et al., 2011; Flores, 2011). These proteins, however, are not present in all DA neurons at early stages during their projection through midbrain and diencephalon (Lin et al., 2005; Hernandez-Montiel et al., 2008; Torigoe et al., 2013), suggesting that other guidance systems may be involved in determining this pathway. In addition, as dopaminergic axons project only after other axonal populations pioneer tracts through the diencephalon (Figdor and Stern, 1993; Mastick and Easter, 1996; Nural and Mastick, 2004), it is possible that preceding axons serve as substrate and provide navigational information for dopaminergic axon growth.

No information exists to date regarding substrate-anchored signals on non-dopaminergic axons that may regulate dopaminergic pathfinding. In this respect, unknown substrate-anchored signals appear to influence rostral growth of mDA axons (Nakamura et al., 2000) and the IgCAM cell adhesion protein L1 is involved in the survival, maintenance, axon extension, and migration of dopaminergic neurons (Poltorak et al., 1992; Hulley et al., 1998; Ohyama et al., 1998; Demyanenko et al., 2001). Moreover, L1, NCAM, and NrCAM, have been detected in embryonic DA axons (Shults and Kimber, 1992; Chao et al., 2003; Schiff et al., 2009).

Since dopaminergic axons are part of the MFB in the adult brain, we tested the hypothesis that the interaction with axons from this tract is relevant for DA axon pathfinding. We determined that DA axons project in close apposition to descending axons of the MFB that express GAD65 and that the latter are necessary for stereotypical DA pathway formation. Moreover, our analysis of Robo1/2 and Slit1/2 double knockout mouse embryos revealed that the DA misprojection observed previously in these mice is accompanied by severe alterations in the GAD65 axon scaffold, which may in part be responsible for the observed DA errors. Additional experiments with cultured embryos and dissociated DA neurons suggest that NCAM and Robo proteins are involved in the interaction of DA axons with their substrate.

## Materials and methods

### Animals

Mice and rats at Universidad Nacional Autónoma de México (UNAM) were handled humanely and killed by cervical dislocation or using CO_2_ with the minimum of distress by qualified personnel in compliance with regulations of the Mexican government regarding the use of laboratory animals for research purposes (NOM-062-ZOO-1999) and following the “Guide for Care and use of laboratory animals” of the Institute of Laboratory Resources, National Research Council, USA. Mice at University of Nevada, Reno (UNR) were maintained according to UNR IACUC protocols, following NIH guidelines. The day of detection of vaginal plug was considered embryonic day 0.5 (E0.5).

Wistar rats were used. The following mouse lines were obtained from The Jackson Laboratories (Bar Harbor, MN): Gad2-IRES-Cre (GAD65^CRE^, Gad2^tm2(cre)Zjh/J^, stock 010802) which expresses the Cre recombinase under that GAD65 promoter (Taniguchi et al., 2011) and ROSA26-DTA (ROSA26^DTA^, B6.129P2-Gt(ROSA)26Sor^tm1/DTA)Lky/J^, stock 009669) which expresses the diphtheria toxin upon cross with a Cre recombinase strain (Voehringer et al., 2008). ROSA26-DTA and Gad2-IRES-Cre mice were maintained as homozygous lines. Gad2-IRES-Cre homozygotes were crossed to CD1 mice and the resulting mice were crossed to ROSA26-DTA homozygotes to obtain embryos carrying both transgenic alleles or only ROSA26^DTA^. Embryos from crosses between four independent male-female pairs were analyzed and the described phenotype was detected in litters from the four pairs. The following primers from Jackson Laboratory catalog were used for genotyping: GAD^CRE^; wild type forward primer number 9981 (5′CTT CTT CCG CAT GGT CAT CT), mutant forward primer number 9983 (5′AAA GCA ATA GCA TCA CAA ATT TCA), and common reverse primer number 9982 (5′CAC CCC ACT GGT TTT GAT TT). ROSA26^DTA^; mutant forward primer number 12211 (5′CCA AAG TCG CTC TGA GTT GTT ATC), mutant reverse primer number 8824 (5′CTC GAG TTT GTC CAA TTA TGT CAC), wild type forward primer number olMR3621 (5′CGT GAT CTG CAA CTC CAG TC), and wild type reverse primer number olMR8546 (5′GGA GCG GGA GAA ATG GAT ATG). For studies with Slit1/2 and Robo1/2 knockout embryos we used lines that were a kind gift from Marc Tessier-Lavigne (Genentech Inc., South San Francisco, CA) (Grieshammer et al., 2004; Long et al., 2004; Lopez-Bendito et al., 2007). Slit1/2 double mutants were offspring of Slit1^−/−^; Slit2^+/−^, interbred with each other for more than 10 generations. Robo1/2 mutant mice were maintained as heterozygotes back-crossed to CD1 outbred wild-type mice for more than 10 generations. PCR genotyping was performed as described elsewhere (Dugan et al., 2011).

### Immunostaining

#### Whole brain staining

Mouse or rat embryos were fixed in 4% of paraformaldehyde (PFA) in phosphate buffer saline (PBS, 10010-056, Molecular probes, Grand Island, NY) for 2–6 h depending on the stage. Fixed embryos were dissected to obtain the whole brain which was washed ten times for 10 min in PBS. The tissue was blocked with 10% goat serum (16210072, Gibco, Life technologies, Grand Island, NY) in PBS for 30 min and washed 10 min with PBS. The primary antibody was diluted in antibody incubation solution [PBS containing 0.3% of Triton X-100 (T8787, Sigma, St. Louis, MO) and 10% goat serum]. Brains were incubated in primary antibodies 16–20 h at 4°C with gentle shaking. The primary antibodies used were: anti-NCAM (Sigma, C9672), anti-NCAM (Sigma, SAB2501672), anti-GAD65 (EMD Millipore, MAB351, Billerica, MA), anti-TH (Pelfreez, P40101-0), anti-GAD67 (Chemicon MAB5406, Temecula, CA), anti-GABA (Sigma, A2052), anti-Robo 1 and 2 (kind gift of Elke Stein, Yale University), anti-Robo 1 (Sigma, WH0006091M1), anti-Robo 2 (Sigma, WH0006092M1), anti-TAG1 (R&D, AF4439, R&D Systems, Minneapolis, MN) and anti-L1 (Millipore, CBL275). After incubation, the samples were washed ten times for 10 min in PBS at room temperature. A second blocking was performed as described above followed by incubation for 2 h in secondary antibody diluted 1:1000 in antibody incubation solution. After ten washes for 10 min each in PBS at room temperature, brains were hemisected by cutting along the dorsal and ventral midlines and mounted flat on glass slides with the pial side facing up in 10% glycerol in PBS containing 0.4 mg/ml DABCO (290734, Sigma). The secondary antibodies used were: Cy3 goat anti-mouse (A10521, Molecular Probes), Cy3 goat anti-rabbit (Molecular probes, A10520), Fluor Alexa 488 goat anti-rabbit (Invitrogen, A11034, Life Technologies, Grand Island, NY), Alexa fluor 488 goat anti-mouse (Invitrogen, A11029), Avidin Alexa fluor 488 conjugate (Invitrogen, A21370), Anti-goat biotinylated (Invitrogen, A21370), and ABC kit standard Vector (Jackson immuno research, PK6100, West Grove, PA).

Frozen sections and cultured cells. Frozen sections (30 μm) were collected on SuperFrost Plus slides (VWR, 48311-703, Radnor, PA) and dried for 4 h; dried samples were washed with PBS1X three times and immunostained by incubation of primary antibody mixed in antibody incubation solution for 12–16 h at 4°C. Samples were washed three times for 10 min in PBS, incubated in secondary antibody mix as described above for 2 h at room temperature, washed three times for 10 min in PBS and mounted with DABCO-glycerol as described above.

### Retrograde neuronal labeling

Retrograde labeling was performed as described (Auclair et al., 1999). Briefly, embryos were pinned on their side in a dish containing a layer of cured clear Sylgard and filled with Ringer solution. Rhodamine-Dextran (3000 M.W., D3308, Molecular Probes) was deposited on the tips of minutien pins (0.05 mm diameter, 26002-10, Fine Science Tools, Foster City, CA) as a droplet of a thick solution of the dye dissolved in water and allowed to dry. The midbrain was exposed and the dye was applied by making a small incision in the neuroepithelium with the dye-laden pins. Labeled embryos were incubated 4 h at 35°C with constant bubbling with a gas mix of 95% oxygen and 5% carbon dioxide. After incubation, embryos were fixed in paraformaldehyde for dissection of the brain for immunostaining or direct visualization.

### Embryo culture

Rat embryos were obtained through a cesarean incision from anesthetized Sprague Dawley pregnant dams as described previously for mouse embryos (De Carlos et al., 1996) and incubated for 24 h at 37°C with an in-shop built rotator device. Three to five embryos were incubated in 5 ml of freshly extracted rat serum or in goat serum (69023, Sigma) containing 1% penicillin-streptomycin mix (15070-063, Gibco) perfused constantly with a gas mix of 95% oxygen/5% carbon dioxide. In control cultures we confirmed that growth in rat or goat serum was comparable. The incubation medium was changed once after 12 h of culture. Embryos were then fixed in 4% PFA in PBS followed by whole brain immunostaining. The following recombinant proteins (all from R&D Systems, Minneapolis, MN), were added to the culture medium as indicated in the results section to a final concentration of 40 ng/ml each: Recombinant rat ROBO I/Fc Chimera (1749-RB-050), Recombinant human ROBO 2/Fc Chimera (3147-RB-050), NCAM-C56 Recombinant human NCAM-I/CD56 (2408-NC-050) or Ig-Fc Recombinant human IgG1 Fc, Human IgG1 (110-Hg-1-100).

### Cell culture

Ventral midbrain was obtained and dissociated as described previously (Tamariz et al., 2010). Dissociated cells were diluted to a concentration of 1 × 10^3^ per ml in DMEM (11995, Gibco) supplemented with 1% penicillin/streptomycin mix and L-glutamax (35050079, Gibco) and seeded onto sterile coverslips (12 mm, GG12, Knitell glass, Braunschweig, Germany) coated previously with poly-D-lysine (P4707, Sigma) and the following proteins as indicated in Figure 8E: Fibronectin (F0895, Sigma), Recombinant rat ROBO I/Fc Chimera, Recombinant human ROBO 2/ Fc Chimera, NCAM-C56 Recombinant human NCAM-I/CD56 or Recombinant human IgG1/Fc chimera. Coverslips were coated by incubation in 24-well dishes with poly-D-lysine (0.2 mg/ml in PBS) for 24 h at 37°C in an incubator with 5% CO_2_, then washed three times for 1 h each with sterile water, incubated with the proteins for 4 h (40 ng/ml each in PBS) at 37°C in an incubator with 5% CO_2_, washed three times again and used immediately for culture. Proteins in solution were added 2 h after the culture was initiated at a concentration of 40 ng/ml as indicated on Figure 8E. After 24 h in culture at 37°C and 5% CO_2_, coverslips were fixed in 4% PFA in PBS and immunostained for TH as described for frozen sections. Additional cultures were performed in which the only difference was that Laminin (L2020, Sigma) was used instead of Fibronectin at a final concentration of 1 μg/ml (Figure 8F).

### Microscopy

Confocal microscopes used were: Zeiss LSM510 META, Zeiss LSM 780 and Nikon Eclipse E600 PCM 2000.

### Statistical analysis

The results of axonal length were analyzed in a completely randomized design using the general linear model procedure (SAS Institute Inc., Cary, NC) with the significance level set at *P* ≤ 0.05. Least square means ± s.e.m. were used to analyze differences between treatments (Figure 8). The results of axon colocalization (Figure 6) were analyzed by a frequencies procedure, the Pearson correlation coefficient was used (SAS Institute Inc., Cary, NC).

## Results

### Ascending dopaminergic axons project in close apposition to early projecting descending GAD65^+^ axon fascicles

**Figure 1:**
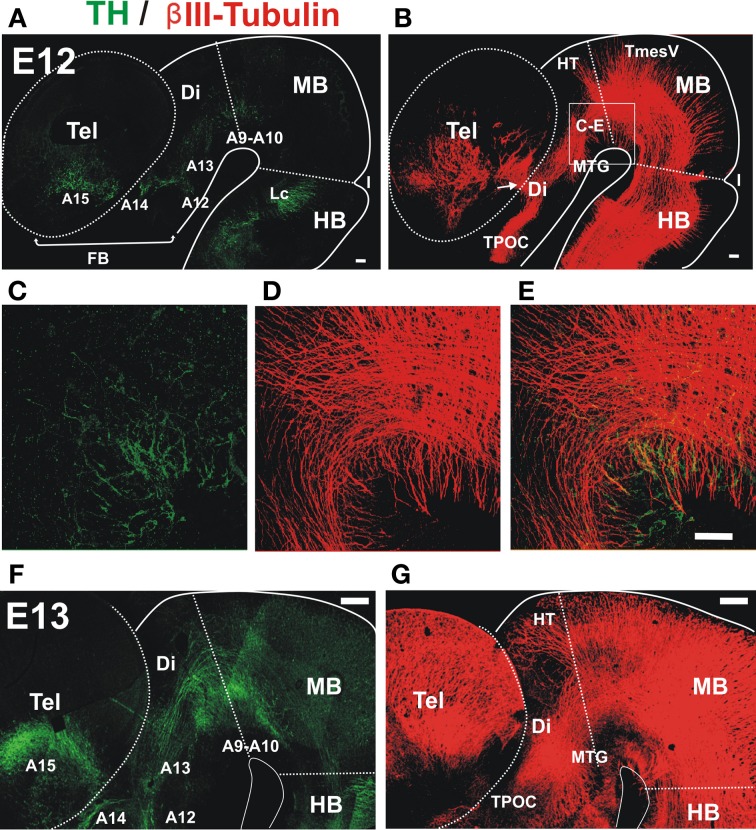
**Early projecting midbrain TH axons grow into territories containing pre-existing non-dopaminergic axon bundles**. Whole brains from E12 **(A–E)** and E13 **(F,G)** rat embryos were immunostained, cut along dorsal and ventral midlines and the resulting hemibrains were mounted flat. Confocal images were collected and are shown here as projections of Z-stacks. **(A,C,E,F)** Tyrosine Hydroxylase (TH, green) immunostaining. Approximate location of dopaminergic cell groups is indicated from A9 to A15. **(B,D,E,G)** β III-Tubulin-stained fiber tracts (red). **(C–E)** Show a magnified view of a field in the ventral MB region and part of the diencephalon (Di) indicated in **(B)**. Arrow in **(B)** indicates the point of entry of TH axons into the striatal anlage. Abbreviations: Tel, Telencephalon; Di, Diencephalon; I, Isthmus; HB, hindbrain; MB, midbrain; Lc, locus ceruleus; TPOC, tract of the post-optic commissure; HT, habenular tract; MTG, mammillotegmental tract; TmesV, mesencephalic nucleus of the trigeminal nerve. Scale bar: 100 μm.

Analysis by immunostaining for Tyrosine hydroxylase (TH) revealed the earliest TH^+^ neurons in the ventral aspect of the embryonic rat midbrain and caudal diencephalon at E12 (Figure 1A). Parallel double immunostaining for the axonal marker β III-tubulin at the same stage revealed a very prominent ventral axon bundle that runs longitudinally through the whole extent of the developing brain (Figure 1B). On a closer view it is possible to observe that the first TH^+^ axons grow into the region containing the non-dopaminergic axon bundles (Figures 1C–E). At E13, dopaminergic axons reached the ventral diencephalon en route to the developing striatum along a territory containing the thick non-dopaminergic axon bundle (Figures 1F,G). On its rostral part, this bundle constitutes the MFB which in the adult brain carries ascending midbrain dopaminergic axons (Nieuwenhuys et al., 1982).

**Figure 2:**
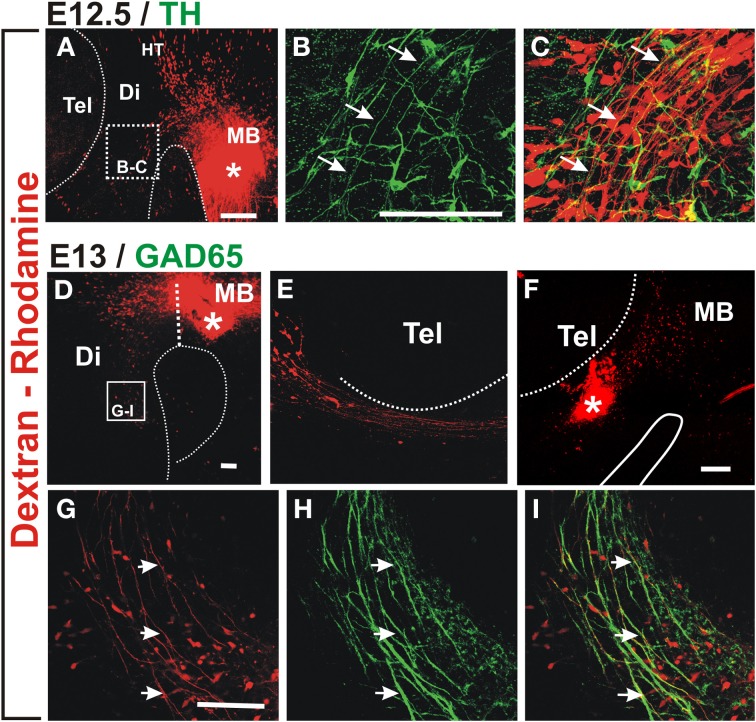
**Retrograde labeling in embryonic rat brain reveals that ascending TH axons interact with descending GAD65 axons**. Dextran-Rhodamine was applied at the locations indicated by asterisks in E12.5 **(A)** and E13 **(D,F)** rat embryos (red) and whole brains were immunostained for TH (**B,C**, green) or GAD65 (**H,I**, green). Flat-mounted hemibrains are shown. **(A,D–F)** are panoramic views; **(B,C)** show a magnified view of the frame indicated in **(A)** revealing apposition of descending Dextran-labeled axons with ascending TH axons (example axon indicated by three arrows). **(F)** shows dextran labeling in the diencephalon at the DA axon entry point into the telencephalon and **(E)** shows the rostral region of the same preparation revealing neurons with descending axons. **(G–I)** are magnified views of the frame indicated in **(D)** and show colocalization of GAD65 signal (green) with all Dextran-labeled axons (red, examples indicated by arrows) consistent with expression of GAD65 in descending axons. Abbreviations as in Figure 1. Scale bars: 100 μm.

To determine the origin of the non-dopaminergic axons that precede dopaminergic axons, we performed axon labeling with Dextran-Rhodamine from the ventral midbrain in E12.5 and 13 embryos which revealed that only descending axons project through this region at this stage (Figures 2A,D). Labeling from a location just caudal to the dopaminergic territory, revealed somata located distantly in the hypothalamic region which likely correspond to neurons of the mammillo-tegmental tract (MTG). At E13, retrograde labeling from the diencephalon at the point of entry of DA axons into the telencephalon revealed neuronal somata in this latter region (Figures 2E,F). These results reveal that this longitudinal axon bundle is in fact composed by segments from different neuronal populations and that no single neuronal population has axons running through its whole extent at E13. By its location, this bundle appears to be an early form of the MFB.

Immunostaining for TH of retrograde labeled E12.5 preparations, indicate that nascent dopaminergic axons project in close apposition to the axons of Dextran labeled neurons (arrows in Figures 2B,C). Therefore, at the onset of their projection, midbrain dopaminergic axons grow in close apposition to preexisting descending longitudinal axons.

From our unpublished observations we knew that, in collagen gel cultures, midbrain dopaminergic axons grow in bundles that also contain GABAergic axons. To assess the possibility that this interaction occurs *in vivo*, we first analyzed whether the observed descending axon bundles contained GAD65^+^ axons. Retrograde labeling from the midbrain as described above combined with immunostaining for GAD65 confirmed that descending axons from the MTG, from the hypothalamic region, and from the telencephalon, express GAD65 (Figures 2G–I and data not shown).

**Figure 3:**
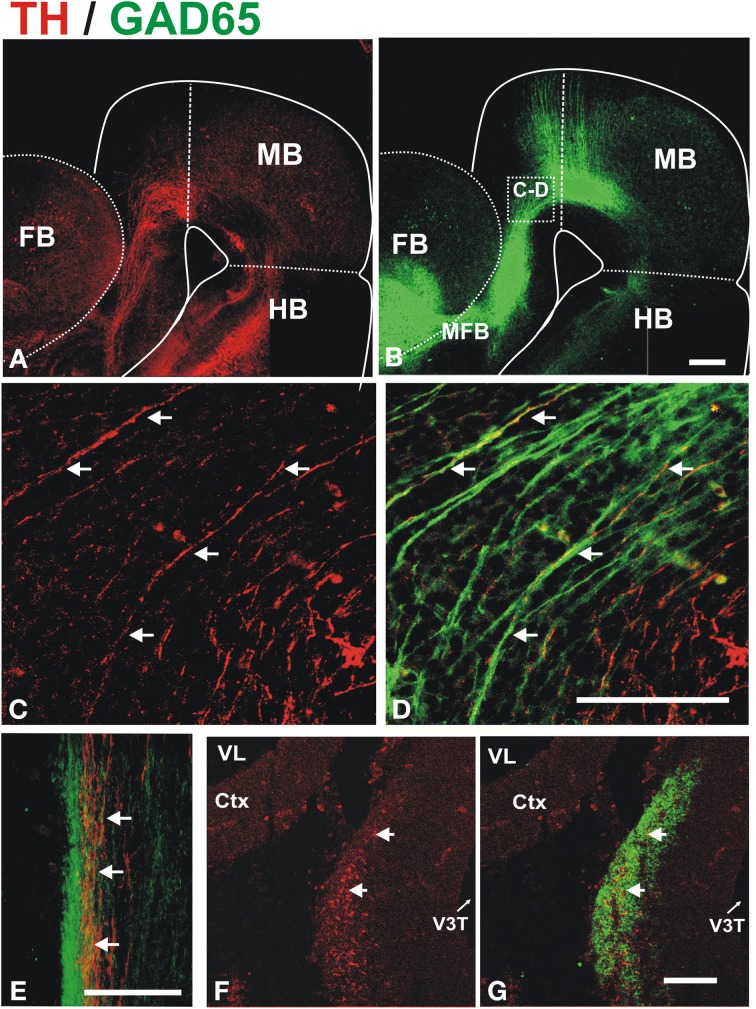
**Dopaminergic axons grow in close apposition to GAD65 axon bundles during mesostriatal projection (E12, E13)**. Whole brains **(A–D)** or frozen sections **(E–G)** from E13 **(A–E)** and E14 **(F,G)** embryonic rat brains were immunostained for TH (red) and GAD65 (green). **(A,B)** Show panoramic views of a flat-mounted hemibrain and the location of magnified field shown in **(C,D)** is indicated in **(B)**. The apposition observed in **(C,D)** was confirmed in single focal planes and in histological sections through the diencephalon in E13 **(E)** and E14 **(F,G)** embryos. Arrows in **(C–G)** indicate TH axons adjacent to GAD65 axons. Scale bar: 100 μm.

To determine whether these descending GAD65^+^ axons interact with growing DA axons, we performed TH/GAD65 double immunostaining of whole E13 and E14 brains (Figure 3). At E13, we observed a prominent GAD65 axon bundle running along the dopaminergic trajectory to the striatum very reminiscent of that observed by β III-tubulin immunostaining (compare Figures 1B, 3B). This observation suggests that many of the axons of the MFB are GAD65^+^. Confocal imaging of the double stained brains revealed that TH^+^ axons grow in close apposition to GAD65^+^ axons along their trajectory in the midbrain, in the diencephalon, and at the entry into the telencephalon (Figures 3C,D and data not shown). Such apposition was also observed in histological transverse sections through the diencephalon at E13 and E14 (Figures 3E–G, respectively). At the later stage, TH axons were found inside the robust longitudinal GAD65 axon bundle (Figures 3F,G).

These results indicate a close apposition of ascending TH^+^ axons with descending GAD65^+^ axons along their trajectory and suggest that, during their growth toward the striatum, dopaminergic axons interact with the various GAD65^+^ axon populations that contribute to the MFB (Nieuwenhuys et al., 1982). This raises the possibility that the GAD65^+^ axons serve as a scaffold for the growth of dopaminergic axons.

### Selective elimination of descending GAD65 axons affects the nigro-striatal projection

To test whether the observed apposition of projecting TH^+^ axons to preexisting GAD65^+^ axons is important for the stereotypical dopaminergic pathfinding we devised a strategy using transgenic mouse lines to eliminate GAD65 axons by the expression of an attenuated form of the diphtheria toxin that ablates developing neurons (DTA). A line expressing the Cre recombinase from the GAD65 locus (GAD65^CRE^) (Taniguchi et al., 2011) was crossed with ROSA26^DTA^ mice (Voehringer et al., 2008). In embryos carrying both alleles, we expected ablation of GAD65^+^ neurons upon activation of the expression of the diphtheria toxin by Cre expressed from the GAD65 gene locus.

**Figure 4:**
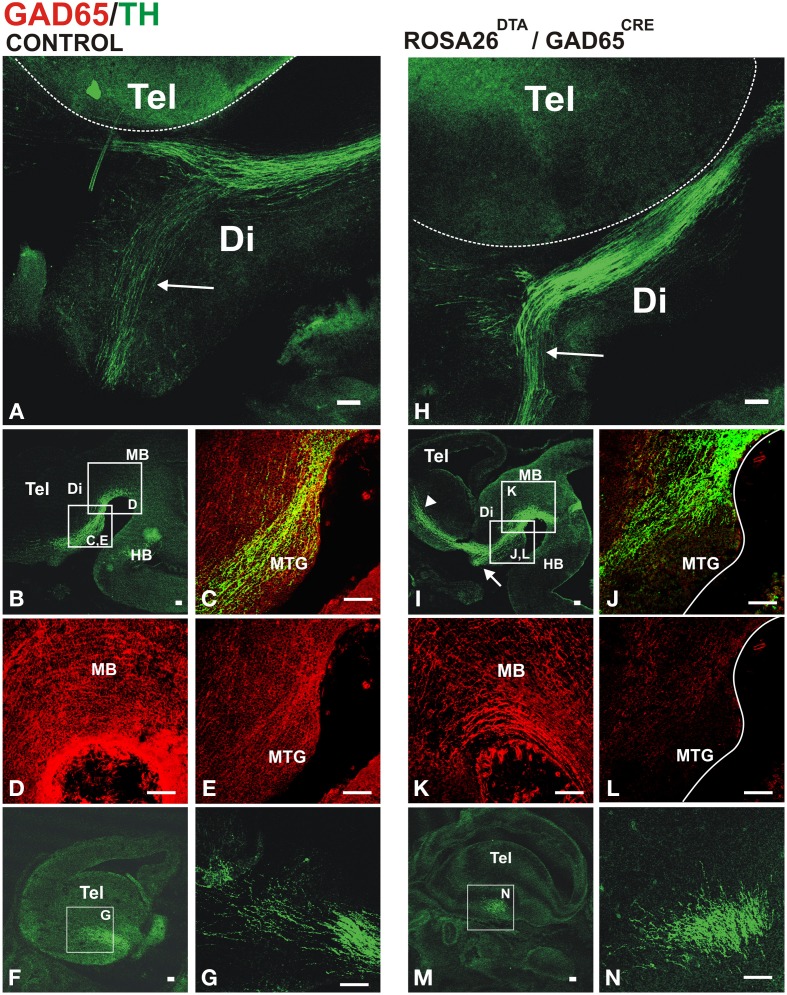
**Conditional depletion of GAD65 axon fibers alters dopaminergic meso-striatal projection**. Brains or sections of E13.5 control mouse embryos **(**ROSA26^DTA/+^, **A–G**) or of embryos carrying both alleles **(**ROSA26^DTA/+^:GAD65^CRE/+^, **H–N)** were immunostained for GAD65 (red) and TH (green). **(A,H)** are views of flat-mounted hemibrains showing the more abundant ventral projection in embryos carrying both alleles (arrows), **(B–E,I–L)** are of parasaggital sections. **(C–E,J–L)** Are magnified views of frames indicated in **(B,I)**, respectively. **(L)** Shows a dramatic reduction in the GAD65 staining compared to **(E)**. **(F,M)** Are parasaggital sections though the telencephalon showing the location of the magnified views in **(G,N)** respectively. Note that the rostro-caudal level in **(F,M)** is different as the location of the TH axons bundle varies between control and double transgenic brains. Scale bar: 100 μm.

Whole brains of E13.5 embryos carrying both transgenic alleles (GAD2^CRE^/ROSA26^DTA^, two embryos from each of four different litters) or control embryos (two wild type CD-1 and six carrying only the ROSA26^DTA^ allele) were immunostained for TH to visualize dopaminergic axon tracts. The normal projection was observed in control embryos as shown in Figures 4A–G. In contrast, embryos carrying both alleles showed misprojection of dopaminergic axons (Figures 4H–N). The TH^+^ axon bundle in these embryos appeared largely normal in the midbrain and diencephalon (Figures 4H,J). Upon reaching the telencephalon in E13, however, many dopaminergic axons failed to veer into the striatal anlage turning instead ventrally into the hypothalamic region (arrows in Figures 4H,I). While the hypothalamic tract was also observed in control embryos, in double transgenic embryos it became a much more robust tract seemingly by the contribution of dopaminergic axons that would normally navigate into the striatum. Moreover, DA axons that entered the striatal anlage overshot rostrally (arrowhead in Figure 4I). On a coronal section, it can also be appreciated that instead of entering the striatum from a medial location as in the control brain (Figures 4F,G), DA axons in the double transgenic mice entered from a ventral location and at a different rostro-caudal level (Figures 4M,N).

To assess the extent of the reduction of GAD65 axons, we performed TH and GAD65 immunostaining on E13.5 control and double transgenic brains. Compared to controls (Figures 4C–E), transgenic GAD65^CRE^/ROSA26^DTA^ embryos (Figures 4J,L), showed a mild reduction in GAD65 immunostaining in the midbrain (Figure 4K) and a more noticeable decrease in the diencephalon (Figure 4L). We interpret this incomplete elimination of GAD65 axons to be the combined result of a protracted generation of new GAD65-expressing neurons and a lag between the onset of Cre expression in those cells and the time in which expression of DTA reaches the effective level that ablates them. Nevertheless, this experiment yielded misprojection results as predicted, thus confirming a role for GAD65^+^ axons on midbrain dopaminergic pathfinding.

Analysis of the dopaminergic projection in the brain of newborn control and double transgenic animals by TH immunostaining (Supplementary Figure 1) revealed in the latter an aberrant spread of DA axons in the diencephalon (arrow in Supplementary Figure 1E) and an abnormal strongly stained domain in the preoptic region (arrow in Supplementary Figure 1D) probably resulting from the ventral misprojection observed in E12.5 embryos. It is important to note that there were no gross abnormalities in the brains of double transgenic animals as assessed in Nissl stained material (Supplementary Figures 1C,F).

### TH^+^ and GAD65^+^ axon fascicles express the Ig superfamily membrane proteins NCAM, Robo1, and Robo2

Our results indicate that dopaminergic axons interact with descending GAD65^+^ axon bundles along their trajectory to the striatum and that this interaction seems to be relevant for their stereotypical pathfinding. To identify molecules that could mediate such interaction, we analyzed the expression of cell adhesion proteins in both axonal groups.

**Figure 5:**
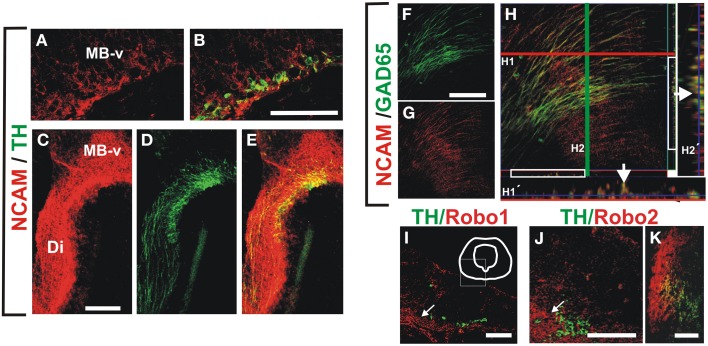
**NCAM is expressed in midbrain dopaminergic and descending GAD65 neurons and non-dopaminergic longitudinal axon bundle expresses Robo proteins**. Whole rat brains or frozen sections were immunostained for NCAM (red) and either TH or GAD65 (both in green). **(A,B)** Show sagittal sections of the ventral midbrain (MB-v) of an E12 embryo. **(C–E)** Are images of a frame in the diencephalon of an E13 embryo. **(F–H)** Are images of a sagittal section in the ventral midbrain region of an E13 brain. **(H1′,H2′)** Are orthogonal views of a confocal Z-stack along the lines (**H1**, red; **H2**, green). Arrows in **(H1′,H2′)** indicate colocalization of GAD65 and NCAM signal. **(I–K)** Coronal sections of rat E12, E13 and E15, were immunostained for TH (green) and either Robo1 or Robo2 (red). **(I,J)** Midbrain (arrows indicate location of longitudinal axon bundle dorsal to the dopaminergic neurons), **(K)** diencephalon. Scale bar: **(A–J)**, 100 μm; **(K)**, 50 μm.

We performed immunostaining for the Ig superfamily proteins L1, TAG-1 and NCAM. Of these proteins only NCAM was detected in patterns that overlapped with GAD65^+^ and TH^+^ axons at the stages analyzed (Figure 5 and data not shown). NCAM expression was detected in ventral midbrain TH^+^ neurons at E12 (Figures 5A,B) and along the dopaminergic trajectory at E13 (Figures 5C–E). Moreover, NCAM was detected in most GAD65^+^ axon fascicles at E13 in the ventral diencephalon (Figures 5F–H).

Since in previous studies Robo1 and Robo2 were shown to have cell adhesion properties (Hivert et al., 2002), and it is known that dopaminergic, MTG, and TPOC axons express these proteins (Tsuchiya et al., 2009; Dugan et al., 2011; Ricano-Cornejo et al., 2011), we analyzed their role in the interaction of dopaminergic axons with other axon types. We confirmed these findings for DA axons (not shown) and observed that the axon bundle dorsal to dopaminergic axons expressed both Robo1 and Robo2 (Figures 5I–K).

These data indicate that NCAM is expressed both in TH and GAD65 axons at the time of dopaminergic pathfinding and that Robo1 and Robo2 are expressed as well in the bundle precursor that we observed is continuous with the MFB and in a fraction of TH neurons from E12 onwards. Hence, these proteins could be involved in the interaction between these axon types during dopaminergic projection.

### Misprojection of midbrain dopaminergic axons in embryos lacking Slit or Robo is accompanied by severe alteration of GAD65^+^ axon scaffolds

**Figure 6:**
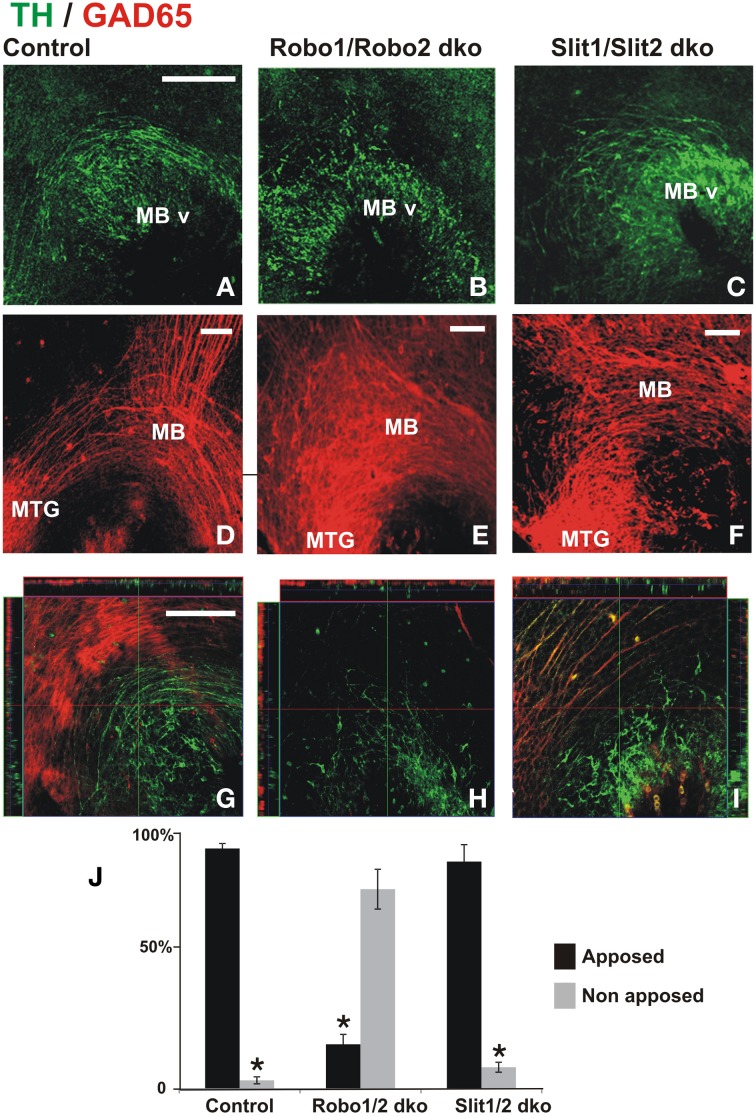
**Dopaminergic misprojection in mouse embryos lacking either Robo1/Robo2 or Slit1/Slit2 is accompanied by dramatic alterations in the GAD65 axon bundles**. Brains from control **(A,D,G)**, Robo1/Robo2 double knock out (dko) **(B,E,H)**, or Slit1/Slit2 dko **(C,F,I)** E12 mouse embryos were double immunostained for TH (green) and GAD65 (red) and hemibrains were mounted flat for observation. Normal projection at this early stage of TH^+^ axon pathfinding can be observed in **(A)** (control). Dramatic reduction and misprojection of TH axons is observed in **(B)** (Robo1/Robo2 dko), and **(C)** (Slit1/Slit2 dko). **(D–F)** Show GAD65 immunostaining of the same areas; in **(D)**, normal GAD65 axon scaffolds are observed while notable alterations in the scaffold are observed in double knockouts (dko) **(E,F)** being more dramatic in Robo1/Robo2 dko **(E)**. **(G–I)** Show single focal planes of the same regions with ortohogonal projections at the red and green lines shown. Apposition of TH axons on the orthogonal projections shown in **(G–I)** was quantified and is shown in **(J)**. Significance (^*^) in **(J)** is *p* < 0.05. Scale bar: 100 μm.

Previous studies have implicated Slit/Robo signaling in dopaminergic axon pathfinding (Bagri et al., 2002; Dugan et al., 2011). Since the studies in the present work revealed the requirement of the GAD65 axon scaffold for normal dopaminergic pathfinding, we formulated the following question: Are dopaminergic misprojections in embryos lacking Robo1/2 accompanied by alterations of the GAD65 axon scaffold? To address that, we analyzed by TH and GAD65 immunostaining whole brains of mutant mouse embryos at a stage in which dopaminergic projection begins. In control embryos we observed the normal DA axon projection into the ventral aspect of the diencephalon and the GAD65 axon bundle running along the ventral aspect of the brain as previously observed in rat embryos (Figures 6A,D,G). As observed before (Dugan et al., 2011) Robo1/2 and Slit1/2 double knockout (dko) embryos had severe dopaminergic projection errors; both types of embryos had a reduction of projection which was more dramatic in Robo mutants (Figures 6B,C) and in addition in Robo1/2 dko embryos there were axons that misprojected dorsally (Figures 6B,H). Furthermore, analysis of GAD65 axons in dko embryos also revealed strikingly disorganized bundles with the effect being more pronounced in Robo1/2 dko embryos (Figures 6E,F). Hence, these results reveal that DA misprojection is accompanied by GAD65 axon scaffold disruption in Robo1/2 and Slit1/2 dko embryos suggesting that at least part of the DA projection errors may result from such underlying GAD65 axon misprojection.

To assess whether the absence of Robo proteins could affect the interaction between TH and GAD65 axons, we analyzed orthogonal planes that were reconstructed digitally from confocal Z-stacks collected from dko preparations. In control and in Slit1/2 dko embryos, we found that most TH axons were apposed to GAD65 axons (Figures 6G,I,J). In Robo1/2 dko embryos, however, most TH axons appeared to grow independent of GAD65 axons (Figures 6H,J). This is clearly appreciated in Figure 6H which is a single focal plane showing mostly TH^+^ axons, as GAD65^+^ axons are largely absent from that plane. These results indicate that interaction between these two axon types is impaired in the absence of Robo1 and Robo2.

### Impairing NCAM and Robo signaling causes misprojection of mesostriatal axons in cultured embryos

With the aim of assessing the role of NCAM and Robo proteins in the nigrostriatal projection we performed whole rat embryo cultures in presence or absence of soluble versions of these proteins consisting only of their extracellular domain which, at least for Robo, are known to interfere with their function (Ricano-Cornejo et al., 2011). After culture for 24 h, embryos were immunostained for TH.

**Figure 7:**
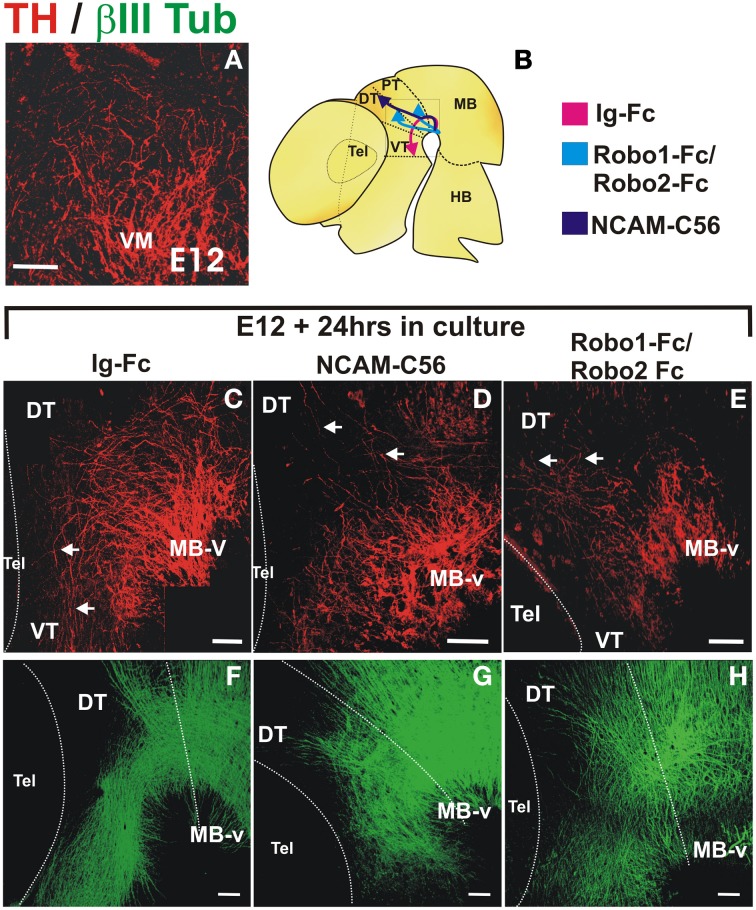
**NCAM and Robo1/2 ectodomains impair projection of midbrain dopaminergic axons in whole embryo culture**. Whole E12 rat embryos were cultured for 24 h in control medium **(C,F)** or in medium with either NCAM or Robo1/Robo2 soluble ectodomains **(D,G**,**E,H**, respectively**)** followed by TH (red) or β III-Tubulin (green) immunostaining. Flat-mounted hemibrains are shown. **(A)** Shows TH signal in a normal E12 embryo at the beginning of the culture. **(C–F)** Show control growth and **(D,G,E,H)** show halted growth or misprojection in cultures with NCAM or Robo1/2 ectodomains, respectively. The diagram in **(B)** shows the growth observed in each condition. Arrows in **(C)** indicate normal growth of TH axons into the diencephalon and in **(D,E)** indicate aberrant dorsally projecting axons. Scale bar: 100 μm.

Cultures were initiated at the onset of dopaminergic axon projection at a stage in which the GAD65^+^ axon scaffold is already present, thus avoiding an earlier and indirect effect as observed in Slit1/2 and Robo1/2 mutant embryos (E12, Figure 7A). In control cultures, a stereotypical projection was observed with numerous axons that turned, projected through the ventral aspect of pretectum (PT) and dorsal thalamus (DT) and reached the ventral thalamus (VT) (*n* = 14) (arrows in Figure 7C). In contrast, in only one of ten cultured embryos with soluble NCAM we observed rostral axon turning, in seven of them there was dorsal projection toward the thalamus reaching the roof plate and in two of them, there were axons projecting caudally toward the hindbrain and some axons reaching the dorsal thalamus (Figure 7D). The most obvious effect in these experiments was the reduction of axons that projected away from the ventral midbrain region. The effect of soluble Robo1 and Robo2 ectodomains, which are expected to impair Robo/Slit function, was more dramatic in the reduction of projection; no axons reached the VT in any of the twelve cultured embryos and in ten of them only dorsal misprojection was detected (Figure 7E). The diagram in Figure 7B shows a summary of the TH growth observed in control cultures and in cultures with Robo and NCAM ectodomains.

Furthermore, immunostaining for β-III tubulin of cultured control embryos revealed overall normal axons scaffolds (Figure 7F). In embryos treated with NCAM, however, β-III tubulin staining showed slight alterations in the thickness of the longitudinal fiber tracts in the midbrain and diencephalon (Figure 7G) while in embryos treated with Robo1/Robo2 axon tracts were more disorganized but with longitudinal axons still present (Figure 7H).

Hence, using Robo/Slit blocking reagents we obtained results that are in keeping with the known role of these proteins on guidance of dopaminergic axons thus supporting the suitability of the soluble NCAM ectodomain as a reagent to interfere with axon projection in culture. Our findings further suggest that NCAM is involved in dopaminergic pathfinding.

### NCAM on a substrate enhances mesencephalic dopaminergic axon growth with the participation of Robo1 and Robo2

**Figure 8:**
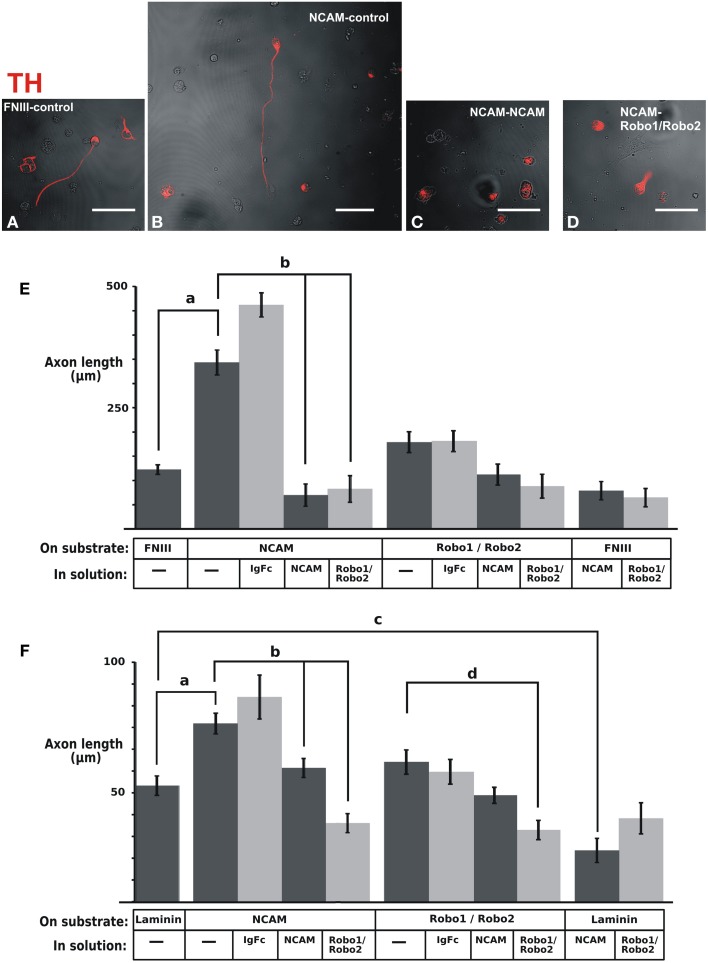
**NCAM on the substrate enhances dopaminergic axon growth in culture and soluble Robo1/Robo2 ectodomains block such effect**. The ventral region of the midbrain containing dopaminergic neurons was extracted from E12 rat brains, dissociated and seeded onto coverslips coated with control substrates [fibronectin (FNIII) or laminin] or the ectodomains indicated. **(A–D)** Show examples of fluorescent TH immunostaining signal (red) overlaid on phase contrast images (b/w) of the same fields of some of the different culture conditions tested. The effect of soluble ectodomains was assessed by adding them to the culture medium as indicated. After culture, TH immunostaining was performed and the length of axons of individual neurons that were not in contact with other axons was quantified. **(E)** Results obtained with fibronectin as control substrate. **(F)** Results obtained with laminin as control substrate. The number of axons quantified for each conditions were as follows. **(E)**: FNIII/control: 261; NCAM/Control: 39; NCAM/Ig-Fc: 38; NCAM/NCAM; 47; NCAM/Robo1&2: 33; Robo1&2/Control: 54; Robo1&2/Ig-Fc: 54; Robo1&2/NCAM: 53; Robo1&2/Robo1&2:41; FNIII/NCAM: 70; FNIII/Robo1&2; 69. **(F)**: Lam/Control: 56; NCAM/Control: 50; NCAM/Ig-Fc: 11; NCAM/NCAM: 58; NCAM/Robo1&2: 58; Robo1&2/Control: 36; Robo1&2/Ig-Fc: 35; Robo1&2/NCAM: 81; Robo1&2/Robo1&2: 58; Lam/NCAM: 36; Lam/Robo1&2: 22. Scale bars: 50 μm.

With the purpose of assessing the potential relevance of NCAM or Robo proteins as mediators of axon interactions, we analyzed whether these proteins could enhance dopaminergic axon growth when presented anchored to the substrate. The ventral midbrain region containing dopaminergic neurons was obtained from E12.5 rat embryos, dissociated and cultured on control glass slides or slides coated with different substrates followed by TH immunostaining. It is important to note that dissociated cultures were performed to identify single TH axons growing independent of any other axons that could affect their interaction with the substrate or their response to the medium components (Figures 8A–D) in contrast with previous studies in which this issue was not considered (Poltorak et al., 1992; Lin et al., 2005; Lin and Isacson, 2006). The first experiments evaluated axon growth on slides coated with fibronectin (FNIII) or on slides coated additionally with the NCAM or Robo1/Robo2 ectodomains (Figure 8E). The additional presence of NCAM on the substrate caused an increase on dopaminergic axon length in comparison with the control growth on FNIII alone (Figure 8E). In contrast, the substrate containing Robo1/Robo2 did not have an effect on axon growth. The specificity of the observed effect of the substrate NCAM was confirmed in cultures in which the medium was additionally supplemented with the soluble form of the protein: the growth increase induced by substrate NCAM was abolished by soluble NCAM (Figure 8E). Surprisingly, however, Robo1/Robo2 ectodomains in the medium also abolished the axon enhancement effect of substrate NCAM (Figure 8E) despite the fact that these proteins failed to induce axon growth when presented on the substrate as described above. Since neither soluble NCAM nor Robo1/Robo2 have any effect on basal growth on the FNIII substrate (last two bars in Figure 8E), this suggests that the effect of substrate NCAM involves the participation of Robo proteins.

A similar, albeit less dramatic, axon growth enhancement of substrate NCAM was observed on an experiment in which Laminin was used as control substrate (Figure 8F). Furthermore, using this substrate we also observed the neutralizing effect of soluble NCAM and Robo1/Robo2 ectodomains upon the growth enhancement induced by substrate NCAM (Figure 8F). It is noteworthy, however, that a decrease in axon length on a Laminin substrate was also induced by soluble NCAM (Figure 8F). Since this suggests that the interaction of the dopaminergic axons with the laminin substrate involves NCAM, the reduction induced by soluble NCAM on the dopaminergic growth on an NCAM substrate may in part result from blocking such interaction.

Overall, these results revealed that NCAM is capable of enhancing dopaminergic axon growth when presented on the substrate and that Robo1 and Robo2 are involved in this effect.

## Discussion

### DA axons require GAD65 axon scaffolds for stereotypical pathfinding

The interaction between axons of different types has been implicated in the establishment of several axon pathways during development. The tract of the postoptic commissure (TPOC) serves as scaffold for axons of the supraoptic tract and of retinal axons (Taylor, 1991; Anderson and Key, 1999) and hindbrain reticulospinal neurons are required for projection of MLF axons that descend from the midbrain (Hernandez-Montiel et al., 2008). Moreover, decussating axons appear to interact with homologous contralateral axons during ventral midline crossing in the hindbrain (Sandoval-Minero and Varela-Echavarria, 2008) and during the formation of the corpus callosum (Fujimori et al., 2000; Rash and Richards, 2001).

In this work we present evidence that precise and long-range navigation of the nigrostriatal projection involves its protracted apposition with different axonal types such as those of the descending GAD65^+^ axons of the MTG and of the MFB primordium. Dopaminergic axons appear to use these tracts as pathfinding scaffold because disrupting GAD65^+^ axon tracts selectively also affects the last choice points along the nigrostriatal route; instead of turning into the telencephalic anlage many DA axons veer ventrally into the hypothalamus along the posterior hypothalamic nucleus (PHT) and others overshoot into rostral regions of the striatum. Hence, our studies reveal a dependence of ascending midbrain DA axons on descending GAD65^+^ axons for their projection to the striatum.

### A possible role for NCAM and Robo1/2 in DA axon interactions

Axon fasciculation may be mediated by IgCAMs which support cell-cell interactions through their extracellular domains containing immunoglobulin and fibronectin motifs. The receptors for axon guidance molecules Robo, Npn, and DCC have structural similarities to cell adhesion proteins and have also been implicated in cell adhesion or neurite outgrowth (Pierceall et al., 1994; Bennett et al., 1997; Shimizu et al., 2000; Hivert et al., 2002; Liu et al., 2004; Martin et al., 2006; Valdembri et al., 2009). Homophilic interactions have been demonstrated for IgCAMs, Npn1, and Robo proteins (Fujisawa et al., 1997; Hivert et al., 2002; Pavlou et al., 2002; Haspel and Grumet, 2003; Liu et al., 2004), and heterophilic interactions occur between Robo1 and Robo2, between different IgCAMs, or between IgCAMs and Npn1 and Npn2 (Morales et al., 1993; Castellani et al., 2000; Liu et al., 2004; Falk et al., 2005; Wright et al., 2007). Consistently, recent findings revealed a role of Npn1 in mediating sensory and motor axon interactions in mouse embryos (Huettl and Huber, 2011; Huettl et al., 2012).

Seeking evidence of the participation of cell adhesion molecules in the interaction between DA and GAD65 axons, we confirmed earlier findings of the presence of NCAM on DA axons (Shults and Kimber, 1992; Schiff et al., 2009) and detected this adhesion molecule in the GAD65 axon bundles as well. Interfering with NCAM function in cultured embryos with soluble NCAM ectodomain caused DA misprojection, and when presented attached to the substrate, this adhesion molecule enhanced DA axon growth. This suggests that NCAM mediates DA axon interaction with the substrate in the developing brain and could be involved in the interaction between DA and GAD65 axons as well.

Since Robo1 and Robo2 are capable of homophilic and heterophilic interactions *in vitro* (Hivert et al., 2002; Liu et al., 2004) and have been proposed to participate in axon interactions *in vivo* (Dugan et al., 2011), we hypothesized that these proteins could support DA growth when presented on the substrate. Our results, nevertheless, failed to confirm this prediction. Surprisingly, however, Robo1/2 ectodomains added to the culture medium abolished the positive effect on axon growth of substrate NCAM. These findings suggest that Robo proteins are involved in the NCAM-mediated interaction of DA axons to the substrate. As NCAM and Robo could act in different manners and on various cellular or axonal substrates, however, further studies will be required to elucidate their precise role on DA axon guidance.

Our findings also led us to test the idea that at least some of the dopaminergic misprojections observed previously in Robo1/2 mutants could be secondary to alterations in the GAD65 axon scaffold, which would also be consistent with the proposed Slit-independent roles of Robo1/2 in axon guidance (Dugan et al., 2011). We analyzed DA and GAD65^+^ axons in Slit1/2 and Robo1/2 double knockout embryos and found that the GAD65^+^ axon tracts were severely altered in both types of mutants. Based on these results we postulate that some of the proposed roles in DA axon pathfinding of Robo and Slit proteins are indirect and involve effects on the GAD65^+^ scaffold.

The additional observation that in Robo1/2 mutants most dopaminergic axons detached from GAD65^+^ axons while in embryos lacking Slit1/2 their apposition was maintained lends further support to the notion of Robo proteins being involved in mediating DA/GAD65 axon interactions.

### Relevance of membrane bound molecules in DA guidance

While our studies reveal a role for interaction with GAD65^+^ axons in DA axon guidance involving Robo proteins and suggest a role of NCAM in such interaction, other proteins anchored to the membranes of these axon types are likely to be involved as well. These proteins may act via homophilic interactions or as heterophilic partners for NCAM or Robo. In this respect, we propose that the role in DA axon growth of other guidance cue cell surface receptors should be revisited to also consider their participation in DA axon interactions with their substrate. Interestingly, in mice lacking Npn2, desfasciculation of DA axons was observed which was attributed to an impaired response to Sema3F (Kolk et al., 2009); the possibility that the lack of Npn2 could also impair the interaction between dopaminergic axons with adjacent fascicles or alter the GAD65^+^ axon scaffold, however, remains unexplored. Moreover, Semaphorin-independent roles of this receptor were implicated in the ipsilateral projection of dopaminergic axons (Torigoe et al., 2013).

Knowledge of the membrane proteins involved in normal dopaminergic pathfinding could allow the design of artificial substrates to favor axon growth of embryonic stem cell-derived DA neurons (esDA) upon intranigral transplant. This could facilitate the development of combination methods involving secreted guidance cues as used recently to elicit axon growth of intranigrally grafted esDA neurons in rats (Diaz-Martinez et al., 2013) along with substrate-anchored proteins to achieve efficient nigrostriatal DA axon growth in Parkinson's disease.

## Author contributions

Claudia M. García-Peña designed and performed experiments and analyzed data. Alfredo Varela-Echavarría and Elisa Tamariz designed experiments and interpreted results. Alfredo Varela-Echavarría and Claudia M. García-Peña wrote the manuscript. All other authors participated in acquisition, analysis or interpretation of data and revised and approved the contents of the manuscript.

### Conflict of interest statement

The authors declare that the research was conducted in the absence of any commercial or financial relationships that could be construed as a potential conflict of interest.

## Supplementary material

Supplementary Figure 1 **Altered projection pattern of TH axons in newborn ROSA26^DTA^**^/+^**:GAD65^CRE^**^/+^ **mice**. Parasaggital sections of P0 mice are shown. **(A,B,D,E)** Show TH immunostaining and **(C,F)** are Nissl-stained sections. Striatum (str), Midbrain (MB). Arrows point to sites where differences between control and double transgenic mice were found. Scale bar, 500 μm.

## References

[B1] Albanese A. Altavista M. C. Rossi P. (1986). Organization of central nervous system dopaminergic pathways. J. Neural Transm. Suppl. 22, 3–17 3465873

[B2] Anderson R. B. Key B. (1999). Role of acetylcholinesterase in the development of axon tracts within the embryonic vertebrate brain. Int. J. Dev. Neurosci. 17, 787–793 10.1016/S0736-5748(99)00064-7 10593614

[B3] Auclair F. Marchand R. Glover J. C. (1999). Regional patterning of reticulospinal and vestibulospinal neurons in the hindbrain of mouse and rat embryos. J. Comp. Neurol. 411, 288–300 10404254 10.1002/(sici)1096-9861(19990823)411:2<288::aid-cne9>3.0.co;2-u

[B4] Bagri A. Marin O. Plump A. S. Mak J. Pleasure S. J. Rubenstein J. L. (2002). Slit proteins prevent midline crossing and determine the dorsoventral position of major axonal pathways in the mammalian forebrain. Neuron 33, 233–248 10.1016/S0896-6273(02)00561-5 11804571

[B5] Bennett K. L. Bradshaw J. Youngman T. Rodgers J. Greenfield B. Aruffo A. (1997). Deleted in colorectal carcinoma (DCC) binds heparin via its fifth fibronectin type III domain. J. Biol. Chem. 272, 26940–26946 10.1074/jbc.272.43.26940 9341129

[B6] Blakely B. D. Bye C. R. Fernando C. V. Prasad A. A. Pasterkamp R. J. Macheda M. L. (2013). Ryk, a receptor regulating wnt5a-mediated neurogenesis and axon morphogenesis of ventral midbrain dopaminergic neurons. Stem Cells Dev. 22, 2132–2144 10.1089/scd.2013.0066 23517308

[B7] Castellani V. Chedotal A. Schachner M. Faivre-Sarrailh C. Rougon G. (2000). Analysis of the L1-deficient mouse phenotype reveals cross-talk between Sema3A and L1 signaling pathways in axonal guidance. Neuron 27, 237–249 10.1016/S0896-6273(00)00033-7 10985345

[B8] Chao C. C. Ma Y. L. Chu K. Y. Lee E. H. (2003). Integrin alphav and NCAM mediate the effects of GDNF on DA neuron survival, outgrowth, DA turnover and motor activity in rats. Neurobiol. Aging 24, 105–116 10.1016/S0197-4580(02)00047-7 12493556

[B9] Dahlstroem A. Fuxe K. (1964). Evidence for the existence of monoamine-containing neurons in the central nervous system. I. Demonstration of monoamines in the cell bodies of brain stem neurons. Acta Physiol. Scand. Suppl. 232, 231–255 14229500

[B10] De Carlos J. A. Lopez-Mascaraque L. Valverde F. (1996). Dynamics of cell migration from the lateral ganglionic eminence in the rat. J. Neurosci. 16, 6146–6156 8815897 10.1523/JNEUROSCI.16-19-06146.1996 PMC6579193

[B11] Demyanenko G. P. Shibata Y. Maness P. F. (2001). Altered distribution of dopaminergic neurons in the brain of L1 null mice. Brain Res. Dev. Brain Res. 126, 21–30 10.1016/S0165-3806(00)00129-2 11172883

[B12] Diaz-Martinez N. E. Tamariz E. Diaz N. F. Garcia-Pena C. M. Varela-Echavarria A. Velasco I. (2013). Recovery from experimental parkinsonism by semaphorin-guided axonal growth of grafted dopamine neurons. Mol. Ther. 21, 1579–1591 10.1038/mt.2013.78 23732989 PMC3734661

[B13] Dugan J. P. Stratton A. Riley H. P. Farmer W. T. Mastick G. S. (2011). Midbrain dopaminergic axons are guided longitudinally through the diencephalon by Slit/Robo signals. Mol. Cell. Neurosci. 46, 347–356 10.1016/j.mcn.2010.11.003 21118670 PMC3021181

[B14] Falk J. Bechara A. Fiore R. Nawabi H. Zhou H. Hoyo-Becerra C. (2005). Dual functional activity of semaphorin 3B is required for positioning the anterior commissure. Neuron 48, 63–75 10.1016/j.neuron.2005.10.024 16202709

[B15] Figdor M. C. Stern C. D. (1993). Segmental organization of embryonic diencephalon. Nature 363, 630–634 10.1038/363630a0 8510755

[B16] Flores C. (2011). Role of netrin-1 in the organization and function of the mesocorticolimbic dopamine system. J. Psychiatry Neurosci. 36, 296–310 10.1503/jpn.100171 21481303 PMC3163646

[B17] Fujimori K. E. Takeuchi K. Yazaki T. Uyemura K. Nojyo Y. Tamamki N. (2000). Expression of L1 and TAG-1 in the corticospinal, callosal, and hippocampal commissural neurons in the developing rat telencephalon as revealed by retrograde and *in situ* hybridization double labeling. J. Comp. Neurol. 417, 275–288 10.1002/(SICI)1096-9861(20000214)417:3<275::AID-CNE2>3.0.CO;2-7[pii] 10683603

[B18] Fujisawa H. Kitsukawa T. Kawakami A. Takagi S. Shimizu M. Hirata T. (1997). Roles of a neuronal cell-surface molecule, neuropilin, in nerve fiber fasciculation and guidance. Cell Tissue Res. 290, 465–470 10.1007/s004410050954 9321711

[B19] Grieshammer U. Le M. Plump A. S. Wang F. Tessier-Lavigne M. Martin G. R. (2004). SLIT2-mediated ROBO2 signaling restricts kidney induction to a single site. Dev. Cell 6, 709–717 10.1016/S1534-5807(04)00108-X 15130495

[B20] Haspel J. Grumet M. (2003). The L1CAM extracellular region: a multi-domain protein with modular and cooperative binding modes. Front. Biosci. 8, s1210–s1225 10.2741/1108 12957823

[B21] Hernandez-Montiel H. L. Tamariz E. Sandoval-Minero M. T. Varela-Echavarria A. (2008). Semaphorins 3A, 3C, and 3F in mesencephalic dopaminergic axon pathfinding. J. Comp. Neurol. 506, 387–397 10.1002/cne.21503 18041777

[B22] Hivert B. Liu Z. Chuang C. Y. Doherty P. Sundaresan V. (2002). Robo1 and Robo2 are homophilic binding molecules that promote axonal growth. Mol. Cell. Neurosci. 21, 534–545 10.1006/mcne.2002.1193 12504588

[B23] Huettl R. E. Haehl T. Huber A. B. (2012). Fasciculation and guidance of spinal motor axons in the absence of FGFR2 signaling. PLoS ONE 7:e41095 10.1371/journal.pone.0041095 22815929 PMC3398880

[B24] Huettl R. E. Huber A. B. (2011). Cranial nerve fasciculation and Schwann cell migration are impaired after loss of Npn-1. Dev. Biol. 359, 230–241 10.1016/j.ydbio.2011.08.019 21925156

[B25] Hulley P. Schachner M. Lubbert H. (1998). L1 neural cell adhesion molecule is a survival factor for fetal dopaminergic neurons. J. Neurosci. Res. 53, 129–134 9671969 10.1002/(SICI)1097-4547(19980715)53:2<129::AID-JNR1>3.0.CO;2-9

[B26] Kolk S. M. Gunput R. A. Tran T. S. Van Den Heuvel D. M. Prasad A. A. Hellemons A. J. (2009). Semaphorin 3F is a bifunctional guidance cue for dopaminergic axons and controls their fasciculation, channeling, rostral growth, and intracortical targeting. J. Neurosci. 29, 12542–12557 10.1523/JNEUROSCI.2521-09.2009 19812329 PMC3097132

[B27] Lin L. Isacson O. (2006). Axonal growth regulation of fetal and embryonic stem cell-derived dopaminergic neurons by Netrin-1 and Slits. Stem Cells 24, 2504–2513 10.1634/stemcells.2006-0119 16840550 PMC2613222

[B28] Lin L. Rao Y. Isacson O. (2005). Netrin-1 and slit-2 regulate and direct neurite growth of ventral midbrain dopaminergic neurons. Mol. Cell. Neurosci. 28, 547–555 10.1016/j.mcn.2004.11.009 15737744

[B29] Liu Z. Patel K. Schmidt H. Andrews W. Pini A. Sundaresan V. (2004). Extracellular Ig domains 1 and 2 of Robo are important for ligand (Slit) binding. Mol. Cell. Neurosci. 26, 232–240 10.1016/j.mcn.2004.01.002 15207848

[B30] Long H. Sabatier C. Ma L. Plump A. Yuan W. Ornitz D. M. (2004). Conserved roles for Slit and Robo proteins in midline commissural axon guidance. Neuron 42, 213–223 10.1016/S0896-6273(04)00179-5 15091338

[B31] Lopez-Bendito G. Flames N. Ma L. Fouquet C. Di Meglio T. Chedotal A. (2007). Robo1 and Robo2 cooperate to control the guidance of major axonal tracts in the mammalian forebrain. J. Neurosci. 27, 3395–3407 10.1523/JNEUROSCI.4605-06.2007 17392456 PMC6672128

[B32] Martin M. Simon-Assmann P. Kedinger M. Martin M. Mangeat P. Real F. X. (2006). DCC regulates cell adhesion in human colon cancer derived HT-29 cells and associates with ezrin. Eur. J. Cell Biol. 85, 769–783 10.1016/j.ejcb.2006.02.013 16762451

[B33] Mastick G. S. Easter S. S. Jr. (1996). Initial organization of neurons and tracts in the embryonic mouse fore- and midbrain. Dev. Biol. 173, 79–94 10.1006/dbio.1996.0008 8575640

[B34] Morales G. Hubert M. Brummendorf T. Treubert U. Tarnok A. Schwarz U. (1993). Induction of axonal growth by heterophilic interactions between the cell surface recognition proteins F11 and Nr-CAM/Bravo. Neuron 11, 1113–1122 10.1016/0896-6273(93)90224-F 8274278

[B35] Nakamura S. Ito Y. Shirasaki R. Murakami F. (2000). Local directional cues control growth polarity of dopaminergic axons along the rostrocaudal axis. J. Neurosci. 20, 4112–4119 10818146 10.1523/JNEUROSCI.20-11-04112.2000 PMC6772636

[B36] Nieuwenhuys R. Geeraedts L. M. Veening J. G. (1982). The medial forebrain bundle of the rat. I. General introduction. J. Comp. Neurol. 206, 49–81 10.1002/cne.902060106 6124562

[B37] Nural H. F. Mastick G. S. (2004). Pax6 guides a relay of pioneer longitudinal axons in the embryonic mouse forebrain. J. Comp. Neurol. 479, 399–409 10.1002/cne.20317 15514979 PMC2080865

[B38] Ohyama K. Kawano H. Asou H. Fukuda T. Oohira A. Uyemura K. (1998). Coordinate expression of L1 and 6B4 proteoglycan/phosphacan is correlated with the migration of mesencephalic dopaminergic neurons in mice. Brain Res. Dev. Brain Res. 107, 219–226 10.1016/S0165-3806(97)00220-4 9593903

[B39] Pavlou O. Theodorakis K. Falk J. Kutsche M. Schachner M. Faivre-Sarrailh C. (2002). Analysis of interactions of the adhesion molecule TAG-1 and its domains with other immunoglobulin superfamily members. Mol. Cell. Neurosci. 20, 367–381 10.1006/mcne.2002.1105 12139915

[B40] Pierceall W. E. Cho K. R. Getzenberg R. H. Reale M. A. Hedrick L. Vogelstein B. (1994). NIH3T3 cells expressing the deleted in colorectal cancer tumor suppressor gene product stimulate neurite outgrowth in rat PC12 pheochromocytoma cells. J. Cell Biol. 124, 1017–1027 10.1083/jcb.124.6.1017 8132705 PMC2119968

[B41] Poltorak M. Shimoda K. Freed W. J. (1992). L1 substrate enhances outgrowth of tyrosine hydroxylase-immunoreactive neurites in mesencephalic cell culture. Exp. Neurol. 117, 176–184 10.1016/0014-4886(92)90124-9 1354165

[B42] Rash B. G. Richards L. J. (2001). A role for cingulate pioneering axons in the development of the corpus callosum. J. Comp. Neurol. 434, 147–157 10.1002/cne.1170 11331522

[B43] Ricano-Cornejo I. Altick A. L. Garcia-Pena C. M. Nural H. F. Echevarria D. Miquelajauregui A. (2011). Slit-Robo signals regulate pioneer axon pathfinding of the tract of the postoptic commissure in the mammalian forebrain. J. Neurosci. Res. 89, 1531–1541 10.1002/jnr.22684 21688288 PMC4128405

[B44] Sandoval-Minero T. Varela-Echavarria A. (2008). Cross-midline interactions between mouse commissural hindbrain axons contribute to their efficient decussation. Dev. Neurobiol. 68, 349–364 10.1002/dneu.20586 18085564

[B45] Schiff M. Weinhold B. Grothe C. Hildebrandt H. (2009). NCAM and polysialyltransferase profiles match dopaminergic marker gene expression but polysialic acid is dispensable for development of the midbrain dopamine system. J. Neurochem. 110, 1661–1673 10.1111/j.1471-4159.2009.06267.x 19619134

[B46] Shimizu M. Murakami Y. Suto F. Fujisawa H. (2000). Determination of cell adhesion sites of neuropilin-1. J. Cell Biol. 148, 1283–1293 10.1083/jcb.148.6.1283 10725340 PMC2174302

[B47] Shults C. W. Kimber T. A. (1992). Mesencephalic dopaminergic cells exhibit increased density of neural cell adhesion molecule and polysialic acid during development. Brain Res. Dev. Brain Res. 65, 161–172 10.1016/0165-3806(92)90175-V 1349268

[B48] Tamariz E. Diaz-Martinez N. E. Diaz N. F. Garcia-Pena C. M. Velasco I. Varela-Echavarria A. (2010). Axon responses of embryonic stem cell-derived dopaminergic neurons to semaphorins 3A and 3C. J. Neurosci. Res. 88, 971–980 10.1002/jnr.22268 19859963 PMC2887602

[B49] Taniguchi H. He M. Wu P. Kim S. Paik R. Sugino K. (2011). A resource of Cre driver lines for genetic targeting of GABAergic neurons in cerebral cortex. Neuron 71, 995–1013 10.1016/j.neuron.2011.07.026 21943598 PMC3779648

[B50] Taylor J. S. (1991). The early development of the frog retinotectal projection. Development (Suppl. 2), 95–104 1842361

[B51] Torigoe M. Yamauchi K. Tamada A. Matsuda I. Aiba A. Castellani V. (2013). Role of neuropilin-2 in the ipsilateral growth of midbrain dopaminergic axons. Eur. J. Neurosci. 37, 1573–1583 10.1111/ejn.12190 23534961

[B52] Tsuchiya R. Takahashi K. Liu F. C. Takahashi H. (2009). Aberrant axonal projections from mammillary bodies in Pax6 mutant mice: possible roles of Netrin-1 and Slit 2 in mammillary projections. J. Neurosci. Res. 87, 1620–1633 10.1002/jnr.21966 19115401

[B53] Valdembri D. Caswell P. T. Anderson K. I. Schwarz J. P. Konig I. Astanina E. (2009). Neuropilin-1/GIPC1 signaling regulates alpha5beta1 integrin traffic and function in endothelial cells. PLoS Biol. 7:e25 10.1371/journal.pbio.1000025 19175293 PMC2631072

[B54] Voehringer D. Liang H. E. Locksley R. M. (2008). Homeostasis and effector function of lymphopenia-induced “memory-like” T cells in constitutively T cell-depleted mice. J. Immunol. 180, 4742–4753 10.4049/jimmunol.180.7.4742 18354198 PMC2670614

[B55] Wright A. G. Demyanenko G. P. Powell A. Schachner M. Enriquez-Barreto L. Tran T. S. (2007). Close homolog of L1 and neuropilin 1 mediate guidance of thalamocortical axons at the ventral telencephalon. J. Neurosci. 27, 13667–13679 10.1523/JNEUROSCI.2888-07.2007 18077678 PMC6673613

[B56] Xu B. Goldman J. S. Rymar V. V. Forget C. Lo P. S. Bull S. J. (2010). Critical roles for the netrin receptor deleted in colorectal cancer in dopaminergic neuronal precursor migration, axon guidance, and axon arborization. Neuroscience 169, 932–949 10.1016/j.neuroscience.2010.05.025 20493932

