# Supplementary figures and images for: Ascending midbrain dopaminergic axons require descending GAD65 axon fascicles for normal pathfinding
Source: Front Neuroanat. 2014 Jun 5;8:43. doi: 10.3389/fnana.2014.00043 (PMC4046268; doi:10.3389/fnana.2014.00043)

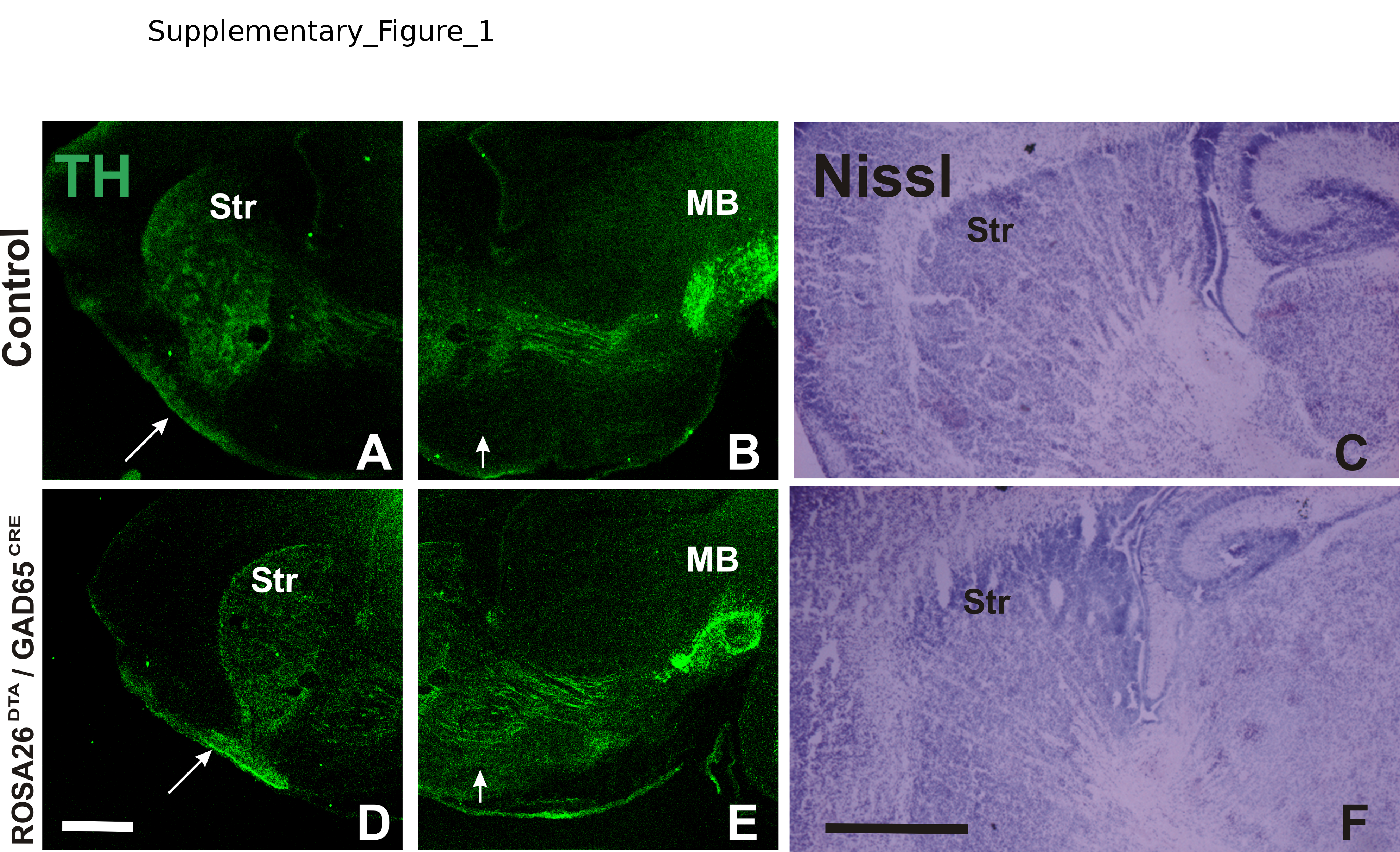

Supplement: Supplementary Figure 1 — Altered projection pattern of TH axons in newborn ROSA26DTA/+:GAD65CRE/+ mice. Parasaggital sections of P0 mice are shown. (A,B,D,E) Show TH immunostaining and (C,F) are Nissl-stained sections. Striatum (str), Midbrain (MB). Arrows point to sites where differences between control and double transgenic mice were found. Scale bar, 500 μm. [file Presentation1.ZIP › Supplementary Figure 1.TIF]
